# Microcrystalline Cellulose Aspiration Presenting as Interstitial Lung Disease Diagnosed via Transbronchial Lung Cryobiopsy

**DOI:** 10.7759/cureus.102026

**Published:** 2026-01-21

**Authors:** Syed M Naqvi, Joshua Wortsman, Hasnain Bawaadam, Sushant Nanavati

**Affiliations:** 1 Pulmonary Medicine, Chicago Medical School, Rosalind Franklin University of Medicine and Science, North Chicago, USA; 2 Interventional Pulmonary and Critical Care Medicine, Aurora Medical Center, Kenosha, USA

**Keywords:** aspiration, foreign body giant cell reaction, interstitial lung disease, microcrystalline cellulose, transbronchial lung cryobiopsy

## Abstract

Chronic occult aspiration is an underrecognized cause of airway-centered and interstitial lung disease and can mimic inflammatory or fibrotic pulmonary disorders. Microcrystalline cellulose, a widely used pharmaceutical excipient, can provoke a foreign body giant cell reaction when aspirated into the lower respiratory tract. Because aspirated material may be focal, it can be missed on small biopsy specimens, delaying diagnosis and leading to inappropriate treatment. We report a case of a 54-year-old woman with progressive exertional hypoxemia and diffuse ground-glass opacities initially concerning for interstitial lung disease. Transbronchial lung cryobiopsy demonstrated polarizable foreign material consistent with microcrystalline cellulose, accompanied by a foreign body giant cell reaction, establishing aspiration-related lung disease. This case emphasizes the importance of considering pill excipient aspiration in patients with recurrent aspiration risk and highlights the diagnostic value of transbronchial lung cryobiopsy in unexplained interstitial lung disease.

## Introduction

Aspiration-related lung disease encompasses a wide spectrum of pulmonary manifestations, ranging from acute aspiration pneumonitis to chronic bronchiolitis and interstitial lung disease. While acute aspiration events are often clinically apparent, chronic occult aspiration frequently goes unrecognized due to nonspecific symptoms, subtle imaging findings, and the absence of a clear aspiration history [1,2]. As a result, aspiration-related lung disease is commonly misclassified as hypersensitivity pneumonitis, infection, sarcoidosis, or idiopathic interstitial pneumonia. Such misclassification has important clinical consequences, as patients may be exposed to prolonged or unnecessary immunosuppressive therapy while the underlying injurious mechanism persists.

Histopathologic confirmation remains the diagnostic gold standard. Characteristic findings include airway-centered inflammation, organizing pneumonia, bronchiolitis obliterans, and foreign body giant cell reactions surrounding exogenous material [3]. However, aspirated material may be sparse or overlooked on conventional transbronchial biopsies, contributing to underdiagnosis [4]. In contrast to intravenous exposure, in which foreign material embolizes to the pulmonary vasculature [5] and produces more diffuse and readily recognizable findings, aspiration-related deposition is often patchy and airway-centered, further limiting clinical recognition.

Microcrystalline cellulose is a purified, partially depolymerized form of cellulose widely used as a filler and binder in pharmaceutical tablets. Pulmonary foreign body reactions to microcrystalline cellulose are classically described in the setting of intravenous injection of crushed oral medications, in which particles embolize to the pulmonary vasculature [5,6]. In contrast, aspiration of microcrystalline cellulose into the airways and alveoli is far less frequently reported and may present with features that closely mimic interstitial lung disease. In this case, transbronchial lung cryobiopsy was critical in establishing the diagnosis by capturing diagnostic airway-centered foreign material with an associated granulomatous reaction.

## Case presentation

A 54-year-old woman was referred for evaluation of chronic exertional hypoxemia, cough, and persistent bilateral diffuse pulmonary infiltrates with ground-glass opacities. Her medical history was significant for diabetes mellitus, gastroesophageal reflux disease, recurrent pneumonia, and known esophageal narrowing or stricture. She had no personal history of autoimmune disease, although her family history was notable for Sjögren’s syndrome in her mother. She was a never-smoker and denied occupational or environmental exposures.

She was hospitalized following an episode of food impaction complicated by vomiting and acute hypoxemic respiratory failure due to aspiration pneumonia, requiring temporary endotracheal intubation for three days. As part of the diagnostic evaluation, upper gastrointestinal endoscopy demonstrated a traversable intrinsic esophageal stenosis with retained food material and reflux-related mucosal changes, supporting impaired esophageal clearance and increased aspiration risk. Bronchoscopy revealed patent airways with moderate to severe proximal bronchial mucosal inflammation and mucus plugging, more pronounced on the right; bronchial washings were obtained without complications.

After discharge, she was evaluated in the outpatient setting and reported overall clinical improvement. During the office visit, she endorsed intermittent chronic cough and persistent dyspnea on exertion but denied fever, hemoptysis, chest pain, or unintentional weight loss. She reported recurrent choking episodes and multiple prior pneumonias, likely related to aspiration events. She continued to require supplemental oxygen, particularly with exertion and during sleep.

Physical examination at the initial outpatient pulmonary evaluation demonstrated mild wheezing on auscultation without digital clubbing, cyanosis, or peripheral edema. Functional assessment with a standardized six-minute walk test demonstrated exertional hypoxemia with associated tachycardia. During home oxygen evaluation, oxygen saturation was 93% at rest on room air and decreased to 88% with ambulation, accompanied by an increase in pulse from 92 to 118 beats per minute. Supplemental oxygen at 2 liters per minute improved resting saturation to 97% and exertional saturation to 92%, although tachycardia persisted with activity. Given the presence of exertional hypoxemia, supplemental oxygen was prescribed for use during physical activity and sleep, while oxygen was not required at rest.

Baseline vital signs and clinical parameters from the initial office visit are summarized separately in Table 1.

**Table 1:** Initial vital signs

Vital sign	Patient value	Reference range
Blood pressure	112/74 mmHg	Systolic <120 mmHg and diastolic <80 mmHg
Heart rate	92 beats/min	60-100 beats/min
Respiratory rate	18 breaths/min	12-20 breaths/min
Body temperature	97 °F (36.1 °C)	97-99 °F (36.1-37.2 °C)
Oxygen saturation	90% on 2 L/min via nasal cannula	95-100% on room air
Body mass index	34.62 kg/m²	18.5-24.9 kg/m²

**Figure 1:**
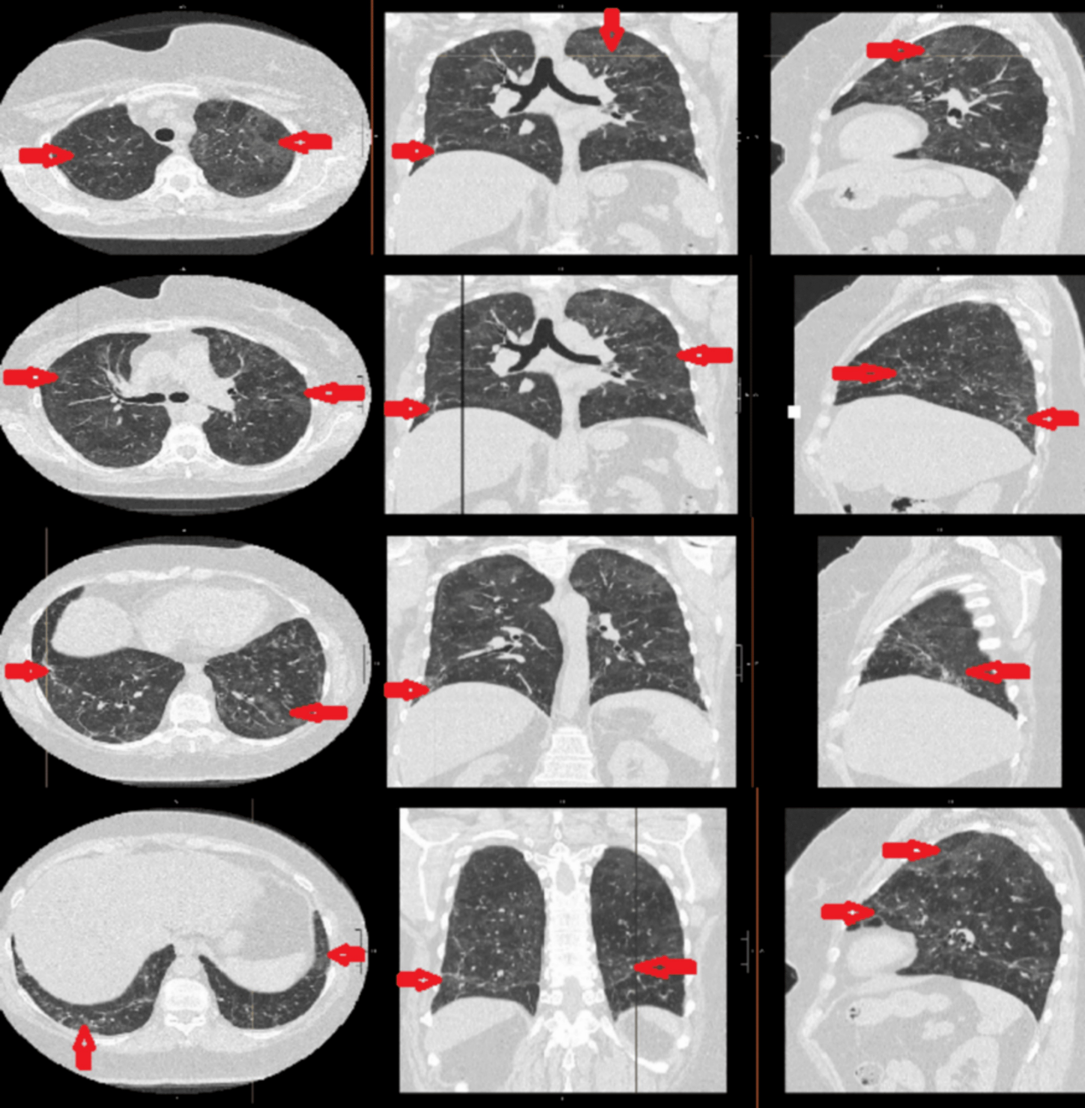
Axial, coronal, and sagittal views of CT of the chest without contrast at four different levels demonstrate diffuse, symmetric lung disease with ground-glass and irregular linear opacities, highlighted by red arrows

CT of the chest without contrast, performed one month after hospitalization, demonstrated diffuse, symmetric lung disease with ground-glass and irregular linear opacities, raising concern for hypersensitivity pneumonitis, interstitial lung disease, drug reaction, or an infectious or inflammatory process (Figure 1).

A serologic evaluation for immune-mediated lung disease was performed and did not support a connective tissue disease-associated or immune-mediated etiology. Anti-neutrophil cytoplasmic antibody testing by indirect immunofluorescence assay was negative, and the antinuclear antibody titer was below the threshold for positivity. An extended autoimmune and myositis antibody panel, as well as hypersensitivity pneumonitis precipitins, was negative. Baseline laboratory evaluation was largely unremarkable, with preserved renal and hepatic function and no evidence of systemic inflammatory, metabolic, or myopathic disease. Mild elevations in bicarbonate and N-terminal pro-B-type natriuretic peptide were considered consistent with chronic hypoxemia and cardiopulmonary stress rather than a primary systemic process. Detailed laboratory results are summarized in Table 2 and Table 3.

**Table 2:** Autoimmune and hypersensitivity pneumonitis serologic evaluation

Test	Result (with units)	Reference range
Hypersensitivity pneumonitis precipitins		
*Aspergillus fumigatus* precipitin	None detected	None detected
*Aspergillus fumigatus* 6 precipitin	None detected	None detected
*Aspergillus flavus* antibody, precipitin	None detected	None detected
*Streptococcus viridans* antibody, precipitin	None detected	None detected
*Thermoactinomyces candidus* antibody, precipitin	None detected	None detected
Antinuclear and connective tissue disease serologies		
Antinuclear antibody, human epithelial type 2 cell line, immunoglobulin G, titer	<1:80	<1:80
Antinuclear antibody screen	0.4	<1.0 index (negative)
Double-stranded deoxyribonucleic acid antibody	1.1 IU/mL	<10 IU/mL (negative)
Smith ribonucleoprotein (extractable nuclear antigen) antibody, immunoglobulin G	3 units	0-19 units
Sjögren syndrome-related antibodies		
Sjögren syndrome-related antigen A 52 (Ro52) (extractable nuclear antigen) antibody, immunoglobulin G	1 AU/mL	0-40 AU/mL
Sjögren syndrome-related antigen A 60 (Ro60) (extractable nuclear antigen) antibody, immunoglobulin G	1 AU/mL	0-40 AU/mL
Sjögren syndrome-related antigen A antibody, immunoglobulin G	<1.0	<1.0 (negative)
Sjögren syndrome-related antigen B antibody, immunoglobulin G	<1.0	<1.0 (negative)
Myositis and overlap antibodies		
Jo-1 (histidyl-transfer ribonucleic acid synthetase) antibody, immunoglobulin G	2 AU/mL	0-40 AU/mL
PL-12 (alanyl-transfer ribonucleic acid synthetase) antibody	Negative	Negative
PL-7 (threonyl-transfer ribonucleic acid synthetase) antibody	Negative	Negative
EJ (glycyl-transfer ribonucleic acid synthetase) antibody	Negative	Negative
OJ (isoleucyl-transfer ribonucleic acid synthetase) antibody	Negative	Negative
Ha (tyrosyl-transfer ribonucleic acid synthetase) antibody	Negative	Negative
Ks (asparaginyl-transfer ribonucleic acid synthetase) antibody	Negative	Negative
Zo (phenylalanyl-transfer ribonucleic acid synthetase) antibody	Negative	Negative
Mi-2 (nuclear helicase protein) antibody	Negative	Negative
Nuclear matrix protein-2 antibody	Negative	Negative
PM/Scl-100 antibody, immunoglobulin G	Negative	Negative
Ku antibody	Negative	Negative
Signal recognition particle antibody	Negative	Negative
P155/140 antibody	Negative	Negative
Transcription intermediary factor 1-gamma (155 kilodalton) antibody	Negative	Negative
Small ubiquitin-like modifier activating enzyme antibody	Negative	Negative
Melanoma differentiation-associated gene 5 (CADM-140) antibody	Negative	Negative
Vasculitis and other antibodies		
Anti-neutrophil cytoplasmic antibody, indirect immunofluorescence assay, titer	<1:20	<1:20
Anti-neutrophil cytoplasmic antibody, indirect immunofluorescence assay, pattern	None detected	None detected
Myeloperoxidase antibody, immunoglobulin G	<1.0	<1.0 (negative)
Serine protease 3 antibody, immunoglobulin G	<1.0	<1.0 (negative)
3-hydroxy-3-methylglutaryl-coenzyme A reductase antibody screen	Negative	Negative

**Table 3:** Baseline hematologic and metabolic laboratory studies

Test	Result (with units)	Reference range
Complete blood count		
White blood cell count	10.8 × 10³/µL	4.5-11.0 × 10³/µL
Hemoglobin	13.0 g/dL	12.1-15.1 g/dL (female), 13.8-17.2 g/dL (male)
Platelet count	284 × 10³/µL	150-450 × 10³/µL
Basic metabolic panel		
Sodium	139 mmol/L	135-145 mmol/L
Potassium	3.8 mmol/L	3.5-5.1 mmol/L
Chloride	103 mmol/L	98-107 mmol/L
Bicarbonate (total carbon dioxide)	30 mmol/L	22-29 mmol/L
Blood urea nitrogen	18 mg/dL	7-20 mg/dL
Creatinine	0.82 mg/dL	0.6-1.1 mg/dL (female), 0.7-1.3 mg/dL (male)
Glucose	91 mg/dL	70-99 mg/dL (fasting)
Calcium	9.6 mg/dL	8.6-10.2 mg/dL
Liver function tests		
Aspartate aminotransferase	12 U/L	8-48 U/L
Alanine aminotransferase	17 U/L	7-55 U/L
Alkaline phosphatase	112 U/L	40-129 U/L
Total bilirubin	0.7 mg/dL	0.1-1.2 mg/dL
Albumin	3.6 g/dL	3.5-5.0 g/dL
Total protein	7.7 g/dL	6.3-7.9 g/dL
Other laboratories		
Creatine kinase	45 U/L	30-220 U/L
N-terminal pro-B-type natriuretic peptide	225 pg/mL	<125 pg/mL (<75 years), <450 pg/mL (≥75 years)

Given the persistence of symptoms, radiographic abnormalities, and diagnostic uncertainty in the setting of known esophageal narrowing and recurrent aspiration events, tissue sampling was pursued. Because of the patient’s comorbidities and prior episode of respiratory failure, a less invasive diagnostic approach was preferred, and transbronchial lung cryobiopsy was sufficient to establish the diagnosis without the risks associated with surgical lung biopsy.

**Figure 2:**
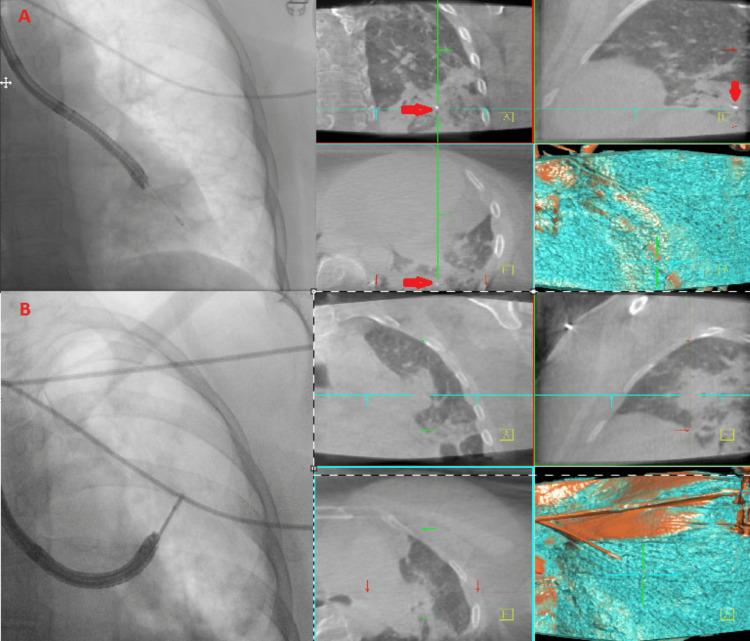
Composite figure illustrating fluoroscopic and cone-beam CT-guided transbronchial lung cryobiopsy sampling from the left lower lobe (A) and left upper lobe (B) In panel (A), red arrows denote the biopsy tool positioned within the target lesion.

Cryobiopsy specimens were obtained from the left lower lobe and left upper lobe to establish a definitive diagnosis (Figure 2).

**Figure 3:**
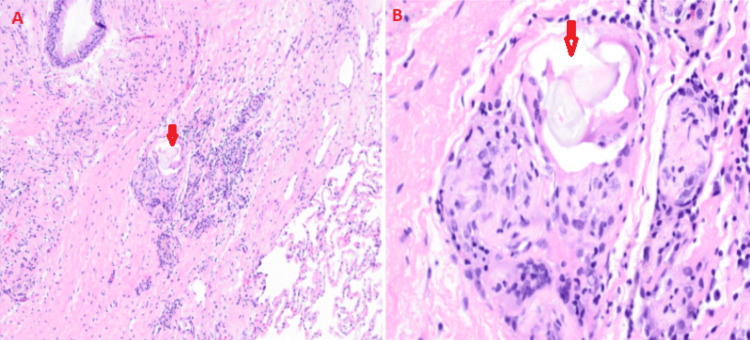
Microscopy of transbronchial cryobiopsy from the left lower lobe of the lung showing aspirated foreign material, including polarizable fragments of microcrystalline cellulose (marked by red arrow), with an associated foreign body giant cell reaction Photomicrographs (A) and (B) are shown at low magnification and high magnification (400x), respectively (H&E stain).

Histopathologic evaluation of the left lower lobe transbronchial lung cryobiopsy specimens demonstrated fragments of benign bronchial wall and scant alveolar parenchyma. Adjacent to the airways, there were collections of aspirated foreign material, including polarizable fragments of microcrystalline cellulose, a common oral medication filler, with an associated foreign body giant cell reaction (Figure 3). No histologic evidence of acute inflammation or neoplasm was observed.

The left upper lobe transbronchial lung biopsy specimen contained a benign fragment of respiratory epithelium without adequate alveolated lung parenchyma for further diagnostic evaluation.

Cytologic examination of bronchial brushing specimens obtained from the right lower lobe was negative for malignant cells. Microscopic review demonstrated a mildly cellular specimen composed predominantly of ciliated bronchial epithelial cells, admixed with mucoid material and alveolar macrophages. Occasional epithelial groups showed uniform nuclear enlargement consistent with reactive changes, reflecting ongoing airway-centered inflammatory injury due to recurrent aspiration. No significant inflammatory infiltrate, granulomas, or malignant features were identified.

The bronchoalveolar lavage (BAL) differential from the right lower lobe is summarized in Table 4. In this case, the mixed inflammatory BAL profile is most consistent with chronic aspiration-related airway inflammation. The elevated neutrophils and lymphocytes, with a relative reduction in macrophages, reflect ongoing airway-centered inflammatory injury rather than a primary immune-mediated interstitial lung disease. Recurrent aspiration events in the setting of esophageal stenosis, impaired esophageal clearance, and severe gastroesophageal reflux provide a unifying mechanism for this pattern, with repeated microaspiration driving neutrophilic inflammation and a secondary lymphocytic response in the distal airways and alveoli.

**Table 4:** BAL differential cell count BAL, bronchoalveolar lavage

Component	Result	Reference range or interpretation
BAL differential		
Neutrophils	29%	≤3%
Lymphocytes	24%	5-15%
Monocytes and macrophages	43%	80-90%
Eosinophils	3%	≤1%
Other cells	1%	Not typically reported in normal BAL
Total cells counted	100	Laboratory dependent

The overall histopathologic pattern supported a diagnosis of aspiration-related lung disease due to microcrystalline cellulose. In the context of the patient’s recurrent aspiration events, esophageal pathology, and gastroesophageal reflux disease, the findings were interpreted as chronic occult aspiration of pill excipients.

A comprehensive gastrointestinal evaluation was performed, including esophagogastroduodenoscopy with endoscopic functional luminal imaging probe assessment and Bravo pH monitoring. Midesophageal biopsies ruled out eosinophilic esophagitis. The gastroesophageal junction was described as Hill grade I. Antral biopsies excluded *Helicobacter pylori*, and duodenal biopsies ruled out celiac disease. Endoscopic functional luminal imaging probe assessment demonstrated decreased esophagogastric junction distensibility. Bravo pH testing performed off proton pump inhibitor therapy showed increased esophageal acid exposure with symptom association. High-resolution esophageal manometry demonstrated an integrated relaxation pressure of 13.8 mmHg with intact peristalsis, including 100% intact swallows and 0% failed swallows. Gastrointestinal findings are summarized in Table 5.

**Table 5:** Gastrointestinal evaluation summary EndoFLIP, endoscopic functional luminal imaging probe

Study	Result	Reference range or interpretation
EndoFLIP		
EndoFLIP at 50 mL	Balloon pressure 41.7 mmHg; esophagogastric junction distensibility index 1.36 mm²/mmHg	Normal esophagogastric junction distensibility index >2.8 mm²/mmHg
EndoFLIP at 60 mL	Balloon pressure 73.7 mmHg; esophagogastric junction distensibility index 1.04 mm²/mmHg	Normal esophagogastric junction distensibility index >2.8 mm²/mmHg
Ambulatory reflux testing		
Bravo pH monitoring off proton pump inhibitor therapy	Acid exposure time 12.2%; supine acid exposure 15.1%; reflux events 145; longest reflux 33.6 minutes; DeMeester score 46.6	Acid exposure time <4% is normal; >6% is abnormal. DeMeester score <14.72 is normal
Symptom association	Heartburn symptom index 90%; symptom association probability 100%	Symptom index >50% is considered positive. Symptom association probability >95% is considered positive
Esophageal motility testing		
High-resolution esophageal manometry	Integrated relaxation pressure 13.8 mmHg; intact swallows 100%; failed swallows 0%	Integrated relaxation pressure upper limit of normal commonly 15 mmHg; intact peristalsis expected in normal motility

Taken together, these findings support a diagnosis of severe gastroesophageal reflux with impaired esophagogastric junction compliance and esophageal clearance, providing a plausible mechanistic explanation for recurrent aspiration and the development of aspiration-related lung disease.

Management priorities shifted toward aspiration risk reduction and reflux control, coordinated with gastroenterology. The patient was counseled on strict aspiration precautions, including eating slowly, avoiding meals close to bedtime, elevating the head of the bed, adhering to reflux-directed therapy, and weight loss as an adjunctive strategy given underlying obesity. She was also treated with a short course of prednisone for inflammatory lung involvement.

On follow-up, the patient reported symptomatic improvement with reduced cough and dyspnea. Repeat oxygen evaluation demonstrated stable oxygen saturations on room air at rest and with exertion, and supplemental oxygen was no longer required. Plans were made for continued outpatient monitoring of symptoms and interval imaging to assess stability following implementation of aspiration risk reduction strategies.

## Discussion

Chronic aspiration of particulate matter is increasingly recognized as an important and underdiagnosed cause of airway-centered and interstitial lung disease. Large clinicopathologic series have demonstrated that aspiration is frequently unsuspected clinically and often diagnosed only after histologic evaluation [3]. Radiologic findings are heterogeneous and commonly misinterpreted as infection, malignancy, or interstitial lung disease [2].

Histologically, aspiration-related lung disease is characterized by bronchiolitis obliterans, organizing pneumonia, and foreign body giant cell reactions surrounding aspirated material [3,7]. While food debris is the most commonly identified aspirated material, pharmaceutical excipients such as microcrystalline cellulose are recognized but uncommon; the present case represents one of these rare presentations.

Several exogenous materials encountered in pulmonary pathology can demonstrate birefringence under polarized light, and recognizing their typical morphology and anatomic distribution helps avoid misclassification. Microcrystalline cellulose appears histologically as rod-like or sheet-like birefringent material under polarized light and may demonstrate a characteristic longitudinal central groove. These features allow distinction from other birefringent particulates, such as talc or calcium oxalate [5]. Histochemical studies have demonstrated consistent staining of microcrystalline cellulose with periodic acid-Schiff, Gomori methenamine silver, Congo red, and modified Russell Movat pentachrome stains, reinforcing diagnostic confidence [8].

Talc, classically associated with inhalational exposure or intravenous injection of crushed tablets, is typically seen as irregular, plate-like birefringent particles and may be accompanied by a granulomatous reaction, often with a vascular or perivascular distribution rather than a purely airway-centered pattern [4]. Calcium oxalate crystals are another important birefringent entity in pulmonary specimens and can occur in association with *Aspergillus *infection (pulmonary oxalosis), typically as strongly birefringent crystals within areas of fungal colonization or necrosis [9]. In aspiration-related lung disease, vegetable or plant matter is more commonly identified than tablet excipients, and clinicopathologic series emphasize that foreign material may be subtle and requires an intentional search with polarized light when aspiration is in the differential diagnosis [3].

Patients with gastroesophageal reflux, esophageal strictures, dysphagia, or impaired swallowing are at increased risk for pill aspiration and aspiration-related lung disease. For patients at increased risk of pill aspiration, practical screening is primarily clinical and prevention-focused rather than protocol-driven. Pill aspiration represents a distinct clinical entity and may be radiographically occult or bronchoscopically subtle, as tablets may dissolve or fragment, leaving only excipient material behind [10]. Iron pill aspiration represents a well-described prototype of this phenomenon, in which dissolution of the active drug leaves behind residual tablet components that incite localized airway injury and foreign body reaction, often leading to delayed or missed diagnosis [11].

Transbronchial lung cryobiopsy has emerged as a valuable diagnostic tool in nonneoplastic lung disease, offering larger tissue samples and improved preservation of lung architecture compared with conventional transbronchial biopsy [4]. This technique facilitates recognition of airway-centered pathology and foreign material and may obviate the need for surgical lung biopsy in selected patients. However, cryobiopsy is associated with procedural risks, including bleeding and pneumothorax, and may be limited by sampling error in patchy disease distributions, underscoring the importance of careful patient selection and performance in experienced centers [12,13].

Recognition of aspiration as the underlying etiology has important therapeutic implications. Management focuses on addressing predisposing factors, including reflux control, evaluation of esophageal pathology, and implementation of aspiration prevention strategies. Failure to recognize aspiration may lead to inappropriate immunosuppressive therapy and progression of lung injury [1,7].

## Conclusions

Microcrystalline cellulose aspiration is a rare but clinically important cause of aspiration-related lung disease that can mimic interstitial lung disease. Transbronchial lung cryobiopsy can be critical for diagnosis by capturing focal foreign material and the associated foreign body giant cell reaction. Clinicians should consider occult aspiration, including aspiration of pill excipients, in patients with diffuse ground-glass opacities and persistent hypoxemia, particularly when serologic evaluation is negative or nonspecific and gastrointestinal risk factors are present.
